# Design and analysis of exoskeleton devices for rehabilitation of distal radius fracture

**DOI:** 10.3389/fnbot.2024.1477232

**Published:** 2024-10-18

**Authors:** Zhiquan Chen, Jiabao Guo, Yishan Liu, Mengqian Tian, Xingsong Wang

**Affiliations:** School of Mechanical Engineering, Southeast University, Nanjing, China

**Keywords:** distal radius fracture, adjustable fixation device, resistance training device, attitude monitoring, surface electromyography, rehabilitation

## Abstract

In this work, the mechanical principles of external fixation and resistance training for the wrist affected by a distal radius fracture (DRF) are revealed. Based on the biomechanical analysis, two wearable exoskeleton devices are proposed to facilitate the DRF rehabilitation progress. Chronologically, the adjustable fixation device (AFD) provides fixed protection and limited mobilization of the fractured wrist in the early stage, while the functional recovery of relevant muscles is achieved by the resistance training device (RTD) in the later stage. According to the designed mechatronic systems of AFD and RTD, the experimental prototypes for these two apparatuses are established. By experiments, the actual motion ranges of AFD are investigated, and the feasibility in monitoring joint angles are validated. Meanwhile, the resistant influences of RTD are analyzed based on the surface electromyography (sEMG) signal features, the results demonstrate that the training-induced muscle strength enhancement is generally increased with the increment in external resistance. The exoskeleton devices presented in this work would be beneficial for the active rehabilitation of patients with DRF.

## 1 Introduction

Distal radius fracture (DRF) is one of the most pandemic injuries in human upper extremity, accounting for one-sixth of attendance in the emergency department and 26% to 46% of all skeletal fractures observed in the primary care setting (Nellans et al., 2012; MacIntyre and Dewan, 2016; Abraham et al., 2009). There are many potential factors (e.g., age, gender, and season) associating with the fracture of radius in distal region, in which the dominant is statistically related to a fall with outstretched arm (MacIntyre and Dewan, 2016; Quadlbauer et al., 2017). In order to protect the head from a violent blow, the primitive motor reflex of extending the hand is naturally evoked when falling, that produces great load transmission along the arm and probably causes the forearm to break at its most weak part—the distal metaphysis of radius (Hove et al., 2014).

Clinically, except for some severe fractures (e.g., comminuted fracture) that demand surgical intervention, the majority of DRF were treated with conservative nonsurgical manner, which mainly involves the closed reduction and immobilization (Sh Ahmed et al., 2020). The rehabilitation of DRF could last for at least several months, and the ultimate purpose is to allow the patient restoring motion, strength and function for activities of daily living (ADLs) (Michlovitz et al., 2001). Surgically, the overall protocol of DRF can be divided into three stages: immobilization, mobilization, and strengthening. The duration of required immobilization varies in patients and treatments, and mobilization is allowed after immobilization continuing for up to about 6 weeks (Valdes, 2009). Minor exercise of the finger, elbow and shoulder joints are strongly suggested when the wrist joint is immobilized, it is a critical to prevent edema occurring in the upper extremity (Ikpeze et al., 2016). To enhance muscle movements during at-home physiotherapy, flexible sensors such as the textile magnetoelastic patch (Xu et al., 2023) were applied for personalized muscle activity monitoring. Combined with machine learning methods, this approach improved understanding of human physiology and offered insights into various health conditions (Xiao et al., 2024).

With the advance of additive manufacturing, the merits of 3D printing aroused great interest from researchers to conduct innovative designs on the external devices for fixation and protection of the broken wrist. The traditionally used plaster cast for the orthotic treatment of wrist, which is heavy, unventilated and hardly removed, has triggered many incidental problems such as the skin diseases and ligament injuries. To increase the comfortableness of wrist orthosis, Kim and Jeong (2015) described a hybrid methodology combining 3D printing and injection molding technology, and significantly reduces the time and cost of manufacturing. To improve the wearing fitness of hand assistive device, Wang et al. (2021) employed a parametric model based on PCA algorithm and 3D printing technology for the fabrication of a customized device that greatly matches the geometry and dimensions of human body. Grski et al. (2019) presented the design and manufacturing process of a personalized wrist orthosis, the contour of upper limb was scanned for CAD design, each part of the orthosis is consisted of two different materials and shaped by 3D printing. Benefited from the advantages of rapid prototyping, cost reduction and manufacturing flexibility, 3D printing technology was extensively adopted for the fabrication of auxiliary medical devices and significantly accelerate the development of rehabilitation research.

Rehabilitation is an indispensable part of the complete protocol of DRF regardless of the fracture type and treatment method. The DRF patients who receive positive rehabilitation by exercise training exhibited more significant improvement on the functionality of wrist, such as the strength of muscles and the range of motion (ROM) (Krischak et al., 2009; Roll and Hardison, 2016). In recent years, an increasing number of researchers commenced investigations on the functional recovery of wrist after fracture, stroke or other diseases affecting the motion ability of upper limb. There are two rotational degrees of freedom (DOFs) for the wrist joint, including flexion/extension (F/E) and radial/ulnar deviation (R/U). According to the typical motion patterns of wrist, Molaei et al. (2022) proposed a robot named MOCH, which was developed by 3D printing with a mass of 1.3 kg. Zhang et al. (2020) presented a parallel wrist rehabilitation robot (PWRR) adopting an open structure to improve the wearable convenience. Andrikopoulos et al. (2015) designed the EXOskeletal WRIST, its motion of robotic appliance was achieved via the pneumatic artificial muscles.

In some cases, the pronation/supination (P/S) of forearm was also considered as a part of the wrist rehabilitation. Based on all these three DOFs (i.e., F/E, R/U, and P/S), Lee and Ben-Tzvi (2017) put forward a forearm exoskeleton for rehabilitative and assistive purposes (FE.RAP), which is relatively lightweight. Martinez et al. (2013) devised a exoskeleton named Wrist Gimbal for the rehabilitation of forearm and wrist, consisting of a serial kinematic structure with three revolute joints that corresponds to the three DOFs. Beekhuis et al. (2013) designed a rehabilitation device for wrist and forearm, it performs dynamically to compensate for misalignment of the upper limb. Oblak et al. (2010) presented a universal haptic drive (UHD) for rehabilitation of either arm or wrist, the desired training mode depends on the selected mechanical configuration on locking/unlocking of a passive universal joint. Shi et al. (2021) developed a cable-driven wrist exoskeleton actuated by the distributed active semi-active (DASA) system, and the performance and effectiveness of this system was experimentally tested.

Meanwhile, some research works paid more attention on the comprehensive rehabilitation of upper limb including wrist and hand. Xiao et al. (2021) introduced a 7-DOF (with only a DOF for the F/E of wrist) wearable hand/wrist exoskeleton for rehabilitation training and real-time motion recognition. Bae and Moon (2012) and Bae et al. (2012) proposed the DULEX-II, a wearable hand robot actuated by three linear actuators and only a DOF for the realization of F/E movement. Meng et al. (2023) designed a soft rehabilitation training glove with the function of safety and personalization. Furthermore, the rehabilitative function was also integrated in many robotic exoskeletons of the upper extremities, such as ARMin I&II (Nef et al., 2006; Mihelj et al., 2007), CADEN-7 (Perry et al., 2007), and MAHI Open Exoskeleton (Dunkelberger et al., 2022).

For years, unremitting efforts have been dedicated to the development and improvement on various assistant apparatus of wrist. Nevertheless, the aforementioned orthotic appliances are simple in structural designs while hardly used for the adjustment of hand. The effectiveness of many axillary exoskeletons of wrist and forearm with two or three DOFs have been verified in previous works, but most of these devices are with relatively heavy weight and complex operation system. As to the exoskeletal robots of upper extremity, most of which are integrated with five to seven DOFs of the entire arm and operated in sophisticated mechatronic platform. Therefore, the current exoskeletons are not applicable to the at-home recovery of DRF in practice. Meanwhile, there are few publications focusing on the resistance-based training of wrist. In general, resistance training is referred to an application of resistance to muscular contraction for building the muscles, it increases the strength of human body by making the muscles work against force (Veldema and Jansen, 2020; Glinsky et al., 2008). Rehabilitation training with sufficient resistance was regarded as an effective and essential way in supporting the functional recovery of muscles.

Given the research status, an adjustable fixation device (AFD) and a resistance training device (RTD), which can be chronologically applied for different rehabilitation stages of DRF, were proposed in this work. In contrast to previous studies that mainly concentrate on the fixation protection of fractured wrists and the passive assistance of wrist motion, the application of AFD and RTD pays more attention to the active rehabilitation of patients suffering from DRF, aiming to reduce muscle rigidity during immobilization and accelerate the recovery progress of wrist motion function. Specifically, the overall research content of this paper is organized as follows: The biomechanical mechanisms of external fixation and resistance exercise for a wrist affected by DRF are described in Section 2, then the mechatronic designs of AFD and RTD are introduced in Section 3, and the application effects of both rehabilitative exoskeleton devices are experimentally examined in Section 4, all the presented contents are discussed and summarized in Section 5.

## 2 Biomechanical analysis

**Figure 1:**
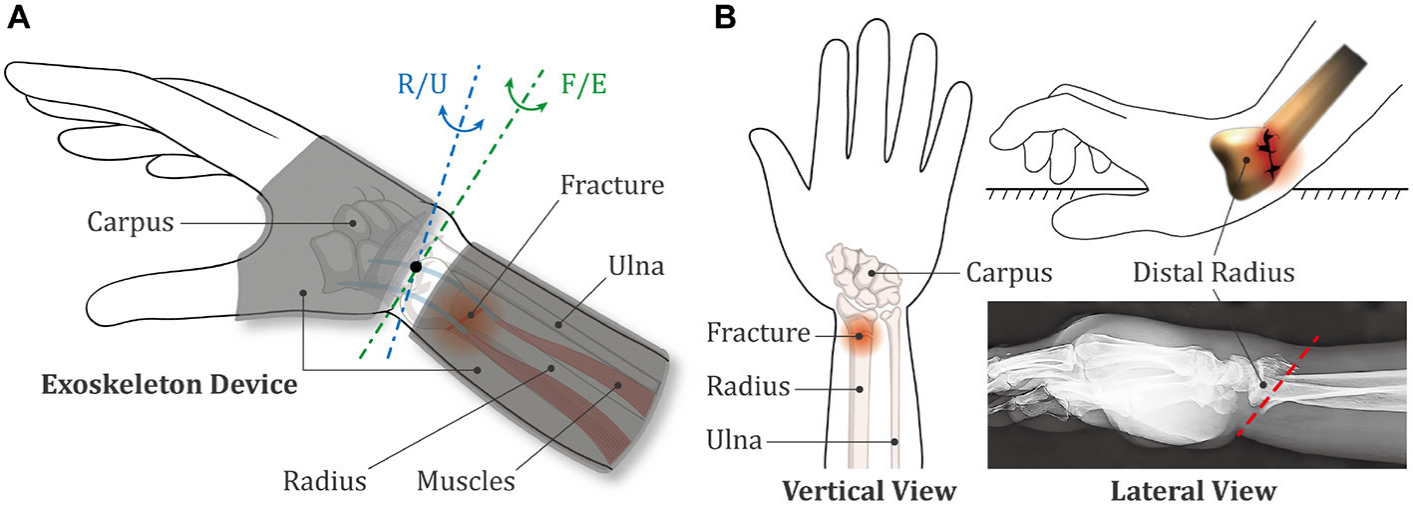
Schematic diagrams of the exoskeleton device and the wrist fracture. **(A)** Positional relationship between the exoskeleton device and the human upper limb. **(B)** Biological characteristics of the Colles fracture.

The relative position between the wearable exoskeleton device and the upper limb is shown in Figure 1A, where the radius is affected by a Colles fracture (CF), which is the most frequent type of wrist fracture and accounts for approximately 90% of all DRFs. To develop the exoskeleton device which is generally applicable, the following analysis were completely carried out based on CF. The human forearm is a complicated system that contains many essential parts, such as bones, muscles, and ligaments. Among all the components, radius and ulna are the two longest bones of forearm, extending from the elbow to the wrist. As presented in Figure 1B, CF is described as a linear transverse fracture of the distal radius with characteristic dorsal tilt, and the broken position is usually located within approximately 2 to 3 cm of the articular surface (Meena et al., 2014).

### 2.1 Mechanism of external fixation

**Figure 2:**
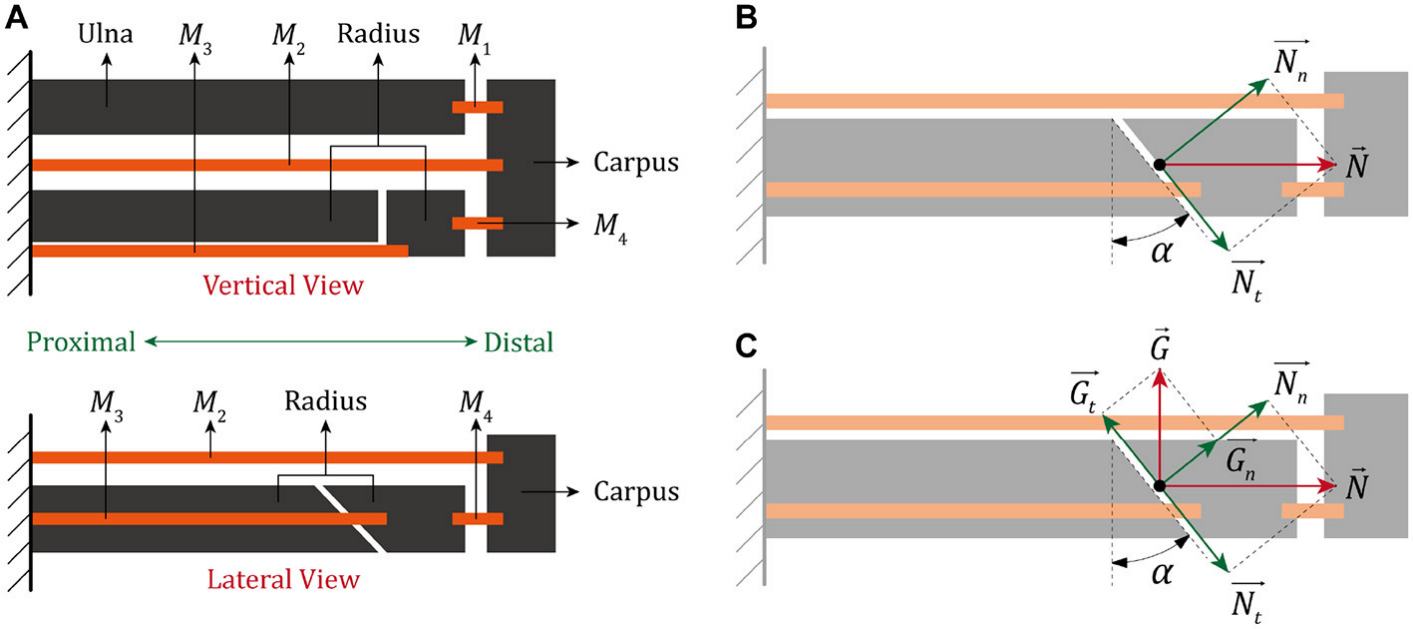
A simplified model of human forearm after the closed reduction treatment of DRF. **(A)** The vertical and lateral view. **(B)** Biomechanical analysis of the fractured end without external fixation. **(C)** Biomechanical analysis of the fractured end with external fixation.

To facilitate understanding, a simplified model of the forearm affected by DRF was established according to the anatomical features of human upper limb. As illustrated in Figure 2A, the proximal part of forearm that closes to the elbow has little impact on the distal fracture, thus this region is basically regarded to be fixed. *M*_1_, *M*_2_, *M*_3_ and *M*_4_ are the muscles and ligament tissues that connect different bones together. In the lateral view, notably, the distal region of radius is separated in a form of dorsal tilt, the same as the distinctive feature of CF.

Based on the presented diagram of DRF, the force received by fracture end is further analyzed. In free condition, as shown in Figure 2B, the clearance of two fracture surfaces is nearly eliminated under the combined action of *M*_3_ and *M*_4_ after the treatment of closed reduction. The contact force between two separated radial bones $\vec{N}$ can be divided into a normal force ($\vec{N_{n}}$) and a tangential force ($\vec{N_{t}}$) in reference to the fracture line, which are given by:


(1) $$\begin{array}{l} \vec{N_{n}}=\vec{N}\cos\alpha \end{array}$$

(2) $$\begin{array}{l} \vec{N_{t}}=\vec{N}\sin\alpha \end{array}$$

where α denotes the inclined angle of fracture line. Theoretically, Equation (3) is supposed to be satisfied if the fractured part of distal radius is able to maintain a position stability without any fixation.


(3) $$\begin{array}{l} N_{t}\leq fN_{n} \end{array}$$

where *f* represents the friction coefficient between two fracture surfaces. By substituting Equations (1, 2) into Equation (3), the stable condition can be further simplified as:


(4) $$\begin{array}{l} \tan\alpha \leq f \end{array}$$

Nevertheless, a large number of environmental factors could potentially affect the maintenance of distal radius and the growth of bone tissues, it is clinically improbable for such a long-term recovery process without any external protection. An auxiliary protector to the wrist is conventionally effective on preventing the closed radial bone from an unexpected re-displacement, here the force caused by fixation and acted on fracture end is equivalent to $\vec{G}$, as shown in Figure 2C.

Subsequently, the resultant forces induced by both the internal tissues and external fixation in the normal and tangential direction can be expressed by Equations (5, 6), respectively.


(5) $$\begin{array}{l} F_{n}=N_{n}+G_{n}=N\cos\alpha +G\sin\alpha \end{array}$$

(6) $$\begin{array}{l} F_{t}=N_{t}-G_{t}=N\sin\alpha -G\cos\alpha \end{array}$$

Therefore, the fundamental requirement for fractured radius to reach a steady fixation state is:


(7) $$\begin{array}{l} N\sin\alpha -G\cos\alpha \leq f\left(N\cos\alpha +G\sin\alpha \right) \end{array}$$

and then the mathematical relationship between the inclined angle of fracture and the applied forces can be deduced as:


(8) $$\begin{array}{l} \tan\alpha \leq \frac{fN+G}{N-fG} \end{array}$$

Here, the maximum inclined angle of fracture end is denoted with α_*max*_, then the following relationship can be obtained:


(9) $$\begin{array}{l} \tan\alpha_{max}=\frac{fN+G}{N-fG}=\frac{f+\frac{G}{N}}{1-f\frac{G}{N}}\geq f \end{array}$$

Compared Equation (5) with Equation (1), it is evident that the action of external fixation increases the overall normal force of fracture end, which correspondingly enhances the friction between fracture surfaces. According to Equations (4, 9), the fracture end is believed to be more stable under the limitation of external fixation, and it is possible for the fractured part to preserve in a steady condition even if the tangent value of fracture angle is larger than the friction coefficient.

According to analysis, the application of external fixation is conducive to prevent the closed fracture radius from another displacement, which is essential for the regeneration of bone tissues. However, the immobilization is not persistently demanded, moderate exercises of wrist are strongly recommended to relieve the muscle stiffness if the recovery of DRF is progressed smoothly (Valdes, 2009; Ikpeze et al., 2016). The requirements on both fixation and motion provide a theoretical basis for the design of assistive device in early rehabilitation stage.

### 2.2 Mechanism of resistance training

Clinically, a long-term limitation to the wrist joint will probably lead to partial loss on the fundamental motion functions for ADLs. Therefore, when the bone tissues are regenerated and the fracture region is basically closed, it is of great importance to further stimulate and enhance the muscle strength. In fact, the resistance training is a favorable practice for rehabilitation and recommended by increased researches. Resistance training is based on the principle that muscles will be naturally activated when they are required to overcome a resistance force, the muscles are getting stronger if patient do the resistant movements repeatedly and consistently. Based on the requirement on simplicity and repeatability, some passive elements are generally adopted for exercise, such as the springs and elastic bands.

**Figure 3:**
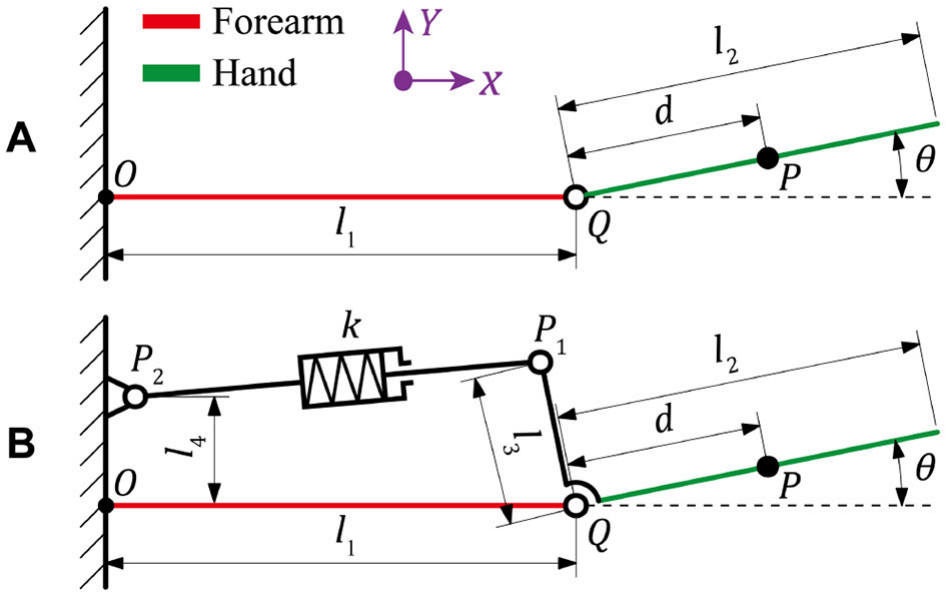
Kinematic diagrams of the wrist movement under **(A)** natural condition and **(B)** resistant condition.

The kinematic models of wrist movement under both the natural and resistant conditions are illustrated in Figure 3, where *O* is the origin of coordinate system, *Q* is the wrist joint, *P* is the centroid of hand, *P*_1_ and *P*_2_ are the fixed points of spring module in hand and forearm, *l*_1_ and *l*_2_ are the lengths of forearm and hand, *l*_3_ is the distance between wrist joint and the fixed end of spring module in hand, *l*_4_ is the distance between forearm and the fixed position of spring module in forearm, *d* is the distance between wrist joint and the hand centroid, *k* is stiffness coefficient of the spring module, and θ is the rotated angle of wrist joint. The mass of entire hand part is set to be *m*, and the coordinates of *P*, *P*_1_, and *P*_2_ are denoted with (*x*_1_, *y*_1_), (*x*_2_, *y*_2_), and (*x*_3_, *y*_3_), respectively. Accordingly, the specific parameters of involved points can be expressed as:


(10) $$\begin{array}{l} \left\{ \begin{array}{c} x_{1}=l_{1}+d\cos\theta \\ y_{1}=d\sin\theta \end{array}\right. \end{array}$$

(11) $$\begin{array}{l} \left\{ \begin{array}{c} x_{2}=l_{1}-l_{3}\sin\theta \\ y_{2}=l_{3}\cos\theta \end{array}\right. \end{array}$$

(12) $$\begin{array}{l} \left\{ \begin{array}{c} x_{3}=0\\ y_{3}=l_{4} \end{array}\right. \end{array}$$

where Equation (10) describes the position characteristics of hand, and the corresponding velocity equation can be further deduced as:


(13) $$\begin{array}{l} \left\{ \begin{array}{c} {ẋ}_{1}=-d\sin\theta \cdot \dot{\theta }\\ {ẏ}_{1}=d\cos\theta \cdot \dot{\theta } \end{array}\right. \end{array}$$

Subsequently, the overall movement velocity *v* is calculated by Equation (14), and the kinetic energy *E*_*k*_ of hand can be obtained, as described in Equation (15).


(14) $$\begin{array}{l} v=\sqrt{{ẋ}_{1}^{2}+{ẏ}_{1}^{2}}=d\dot{\theta } \end{array}$$

(15) $$\begin{array}{l} E_{k}=\frac{1}{2}mv^{2}=\frac{1}{2}md^{2}{\dot{\theta }}^{2} \end{array}$$

In addition, the gravitational potential energy *E*_*p*1_ is expressed as follows:


(16) $$\begin{array}{l} E_{p1}=mgd\sin\theta \end{array}$$

while the other one in this system, the elastic potential energy related to spring module, is given by:


(17) $$\begin{array}{l} E_{p2}=\frac{1}{2}k\text{Δ}x^{2} \end{array}$$

where Δ*x* is the length increment of spring and can be expressed as:


(18) $$\begin{array}{l} \text{Δ}x=\sqrt{{\left(x_{2}-x_{3}\right)}^{2}+{\left(y_{2}-y_{3}\right)}^{2}}-\sqrt{{\left(x_{20}-x_{30}\right)}^{2}+{\left(y_{20}-y_{30}\right)}^{2}}\\ \;=\sqrt{l_{1}^{2}+l_{3}^{2}+l_{4}^{2}-2l_{1}l_{3}\sin\theta -2l_{3}l_{4}\cos\theta }-\sqrt{l_{1}^{2}+{\left(l_{3}-l_{4}\right)}^{2}} \end{array}$$

where *x*_20_, *y*_20_, *x*_30_, and *y*_30_ are the initial coordinate values under the condition of *θ* = 0.

For any mechanical systems, conventionally, the Lagrangian mechanics can be described as the difference between kinetic and potential energies. According to previous discussion, the entire biomechanical system of wrist under a free condition involves only the kinetic energy and the gravitational potential energy, then the Lagrangian function of which is established as follows:


(19) $$\begin{array}{l} L_{1}=E_{k}-E_{p1}=\frac{1}{2}md^{2}{\dot{\theta }}^{2}-mgd\sin\theta \end{array}$$

and then based on the principle of Lagrangian dynamics, the torque of wrist joint *T*_1_ can be obtained as:


(20) $$\begin{array}{l} T_{1}=\frac{d}{dt}\left(\frac{\partial L_{1}}{\partial \dot{\theta }}\right)-\frac{\partial L_{1}}{\partial \theta }=md^{2}\ddot{\theta }+mgd\cos\theta \end{array}$$

Furthermore, by taking the resistant effect of external device into consideration, the Lagrangian function is re-established as follows:


(21) $$\begin{array}{l} L_{2}=E_{k}-E_{p1}-E_{p2}=\frac{1}{2}md^{2}{\dot{\theta }}^{2}-mgd\sin\theta -\frac{1}{2}\\ \;k{\left(\sqrt{l_{1}^{2}+l_{3}^{2}+l_{4}^{2}-2l_{1}l_{3}\sin\theta -2l_{3}l_{4}\cos\theta }-\sqrt{l_{1}^{2}+{\left(l_{3}-l_{4}\right)}^{2}}\right)}^{2} \end{array}$$

and the torque of wrist joint *T*_2_ can be obtained as:


(22) $$\begin{array}{l} T_{2}=\frac{d}{dt}\left(\frac{\partial L_{2}}{\partial \dot{\theta }}\right)-\frac{\partial L_{2}}{\partial \theta }=md^{2}\ddot{\theta }+mgd\cos\theta +T_{e} \end{array}$$

where *T*_*e*_ is calculated by:


(23) $$\begin{array}{l} \begin{array}{l} T_{e}\\ =k\left(\sqrt{l_{1}^{2}+l_{3}^{2}+l_{4}^{2}-2l_{1}l_{3}\sin\theta -2l_{3}l_{4}\cos\theta }-\sqrt{l_{1}^{2}+{\left(l_{3}-l_{4}\right)}^{2}}\right) \end{array}\\ \cdot \frac{1}{2}{\left(l_{1}^{2}+l_{3}^{2}+l_{4}^{2}-2l_{1}l_{3}\sin\theta -2l_{3}l_{4}\cos\theta \right)}^{\frac{-1}{2}}\\ \begin{array}{l} \cdot \left(-2l_{1}l_{3}\cos\theta +2l_{3}l_{4}\sin\theta \right) \end{array} \end{array}$$

Compared Equation (22) with Equation (20), it is evident that an extra torque *T*_*e*_ is generated under the application of spring resistance. To overcome a larger torque in the wrist joint, the muscles of forearm are required to consume more energies, which could ultimately contribute to increased strength and improved motion ability.

## 3 Mechatronic system design

In general, the anatomically available ROM for wrist flexion and extension is 75° and 70°, and for radial and ulnar deviation is 20° and 35°, respectively. Intervention schemes for wrist rehabilitation are conventionally directed toward the acquisition of maximum ROM, while it is hardly possible to realize in the short term, especially for the patients with severe fractures. As a consequence, the required minimum range of motion for comfortably and effectively performing ADLs, also known as the functional ROM, was usually introduced to evaluate recovery effect of the broken wrist in early stage. The functional ROM of each wrist motion is less than the anatomical ROM, namely 40° for both the flexion and extension, 10° and 20° for the radial and ulnar deviation, respectively. The motion characteristics of wrist joint provide a theoretical foundation for the development of rehabilitation devices.

### 3.1 Adjustable fixation device

**Figure 4:**
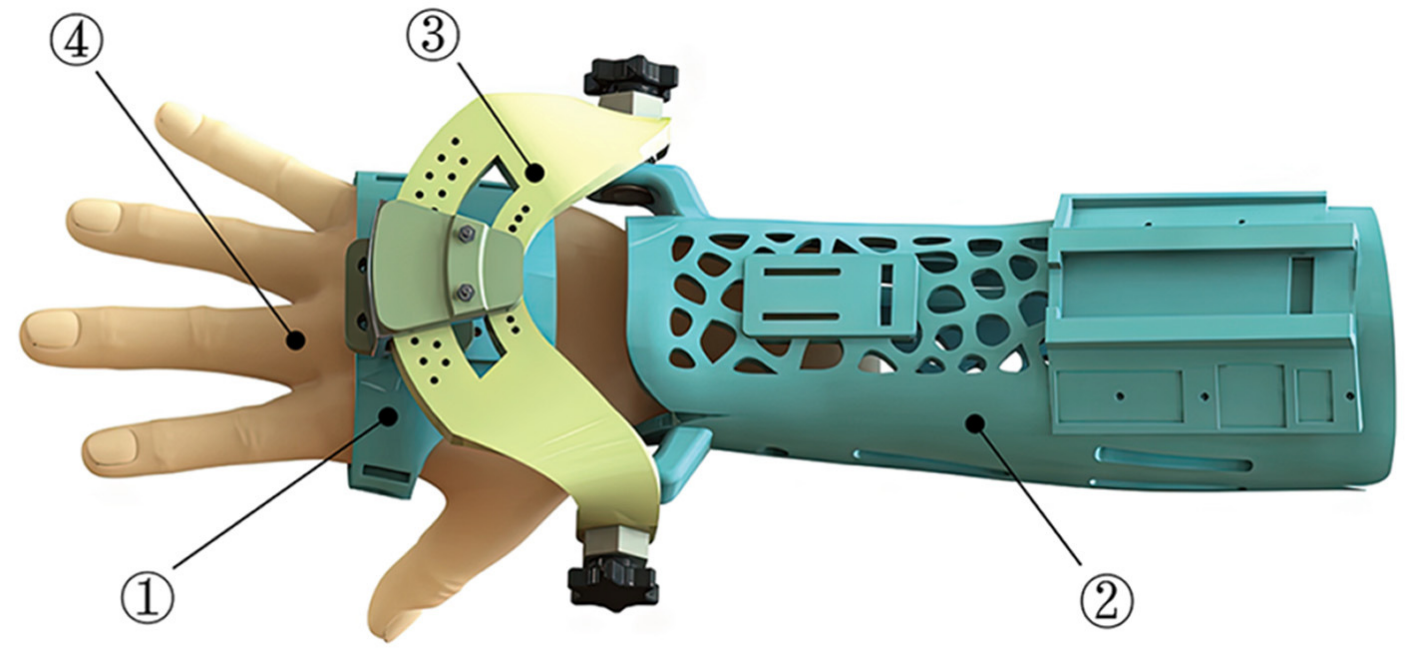
The composition and wearing effect of AFD (1-Hand module; 2-Forearm module; 3-Wrist module; 4-Hand model).

As presented in Figure 4, the designed AFD adopts a modularized structure, which is mainly comprised of three independent integrated parts. The two wearable modules of hand and forearm can be detached and substituted for personal customization, while the intermediate wrist module connects them together and is adjustable for basic wrist movement within the functional ROM.

**Figure 5:**
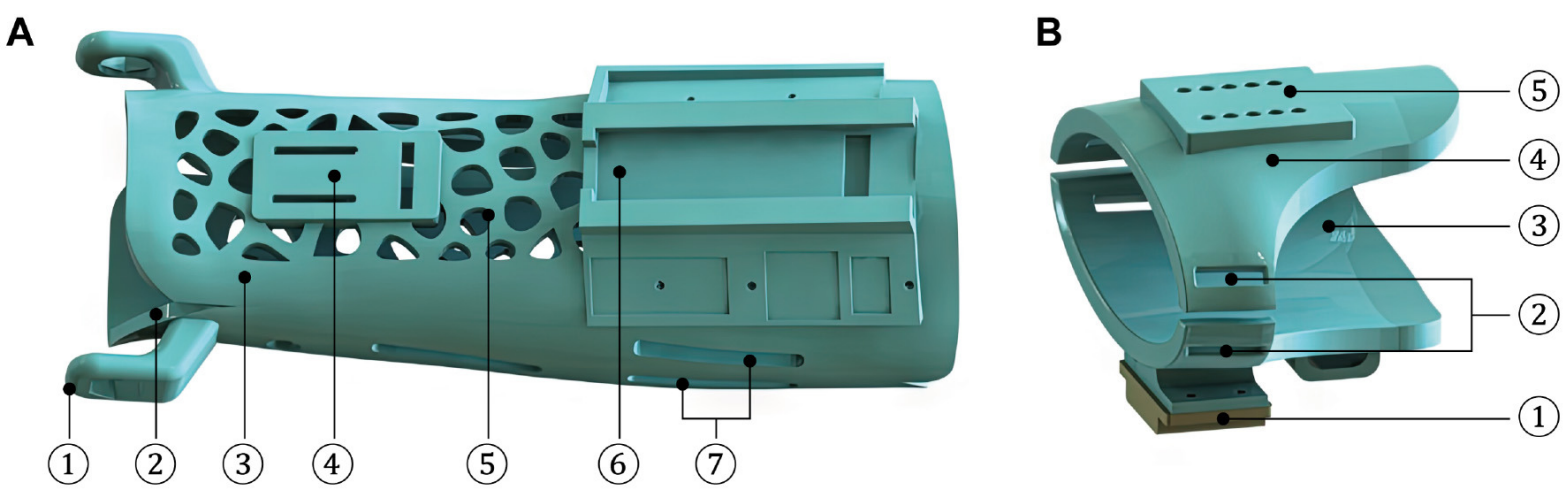
Structures and compositions of the wearing parts of AFD. **(A)** The forearm module (1-Rotation center of R/U movement; 2-Ventral component; 3-Dorsal component; 4-Installation platform of temperature sensor; 5-Ventilation hole; 6-Installation platform of IMU sensor; and 7-Velcro hole). **(B)** The hand module (1-Installation platform of IMU sensor; 2-Velcro hole; 3-Palmar component; 4-Dorsal component; and 5-Location hole).

The structural design and compositions of forearm and hand modules are shown in Figure 5. For the forearm module, the internal surfaces of ventral and dorsal components, which directly contacts with the human forearm, are shaped based on the acquired and processed 3D scanning contours of the right forearm of experimenter. This kind of reverse engineering was presented in many previous literature (Grski et al., 2019; Lin et al., 2016; Asanovic et al., 2019). The application of Voronoi holes not only improves the quality attribute and external appearance of AFD, but also contributes to diminish the possibility of dermatosis triggered by long-term wearing process to a certain extent. In addition, two platforms are designed for the installation and deployment of sensors, and the Velcro holes are used to fasten the ventral and dorsal components together with Velcro types. As to the hand module, the palmar part is equipped with the other installation platform of posture sensor, and the Velcro holes are configured for closed constraint between the palmar and dorsal components.

**Figure 6:**
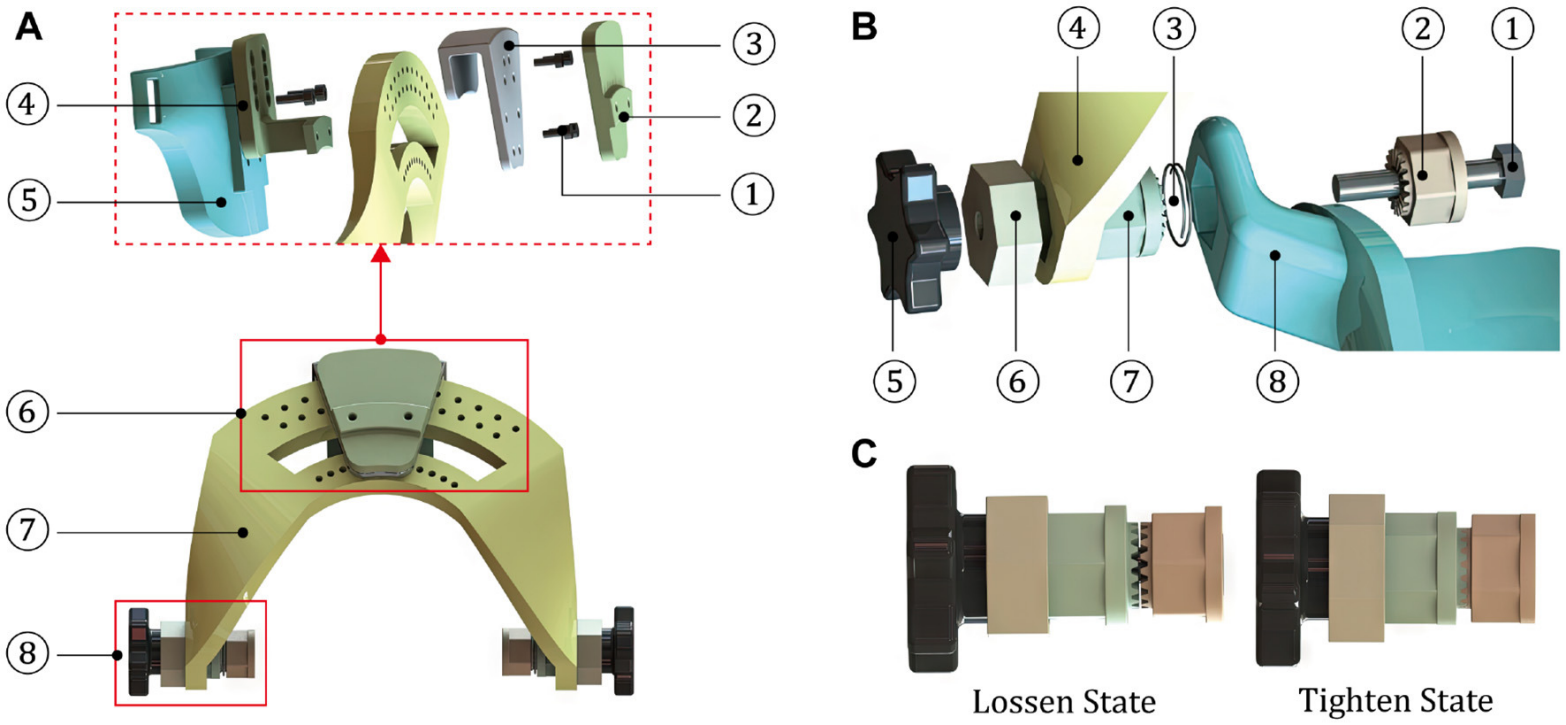
Structures and compositions of the motion and connection parts. **(A)** The wrist module (1-Screw; 2-Covering cap; 3-Slider connector; 4-Trajectory slider; 5-Dorsal component of the hand module; 6-R/U adjusting mechanism; 7-Arc element; and 8-F/E adjusting mechanism). **(B)** The F/E adjusting mechanism (1-Hexagon bolt; 2-Part 1 of the toothed connector; 3-Spring; 4-Arc element of the wrist module; 5-Rotary handle; 6-Axial limiting ring; 7-Part 2 of the toothed connector; and 8-Ventral component of the forearm module). **(C)** Two states of the F/E adjusting mechanism.

As shown in Figure 6, the wrist module is an intermediate part that integrates the hand and forearm modules into a coherent mechanical system, in which the two auxiliary rotation DOFs are achieved by the R/U and F/E adjusting mechanisms. The dorsal component of hand module is fixed with a trajectory slider of R/U mechanism, and the designed range of R/U movement is limited by an arc element. As for the F/E mechanism, it is configured to share an identical axis with the arc connector and the ventral component of forearm module. The rotary degree of F/E movement can be manually regulated under the loosen state, while the fixation of certain F/E posture is supposed to be configured as the tighten condition.

### 3.2 Resistance training device

**Figure 7:**
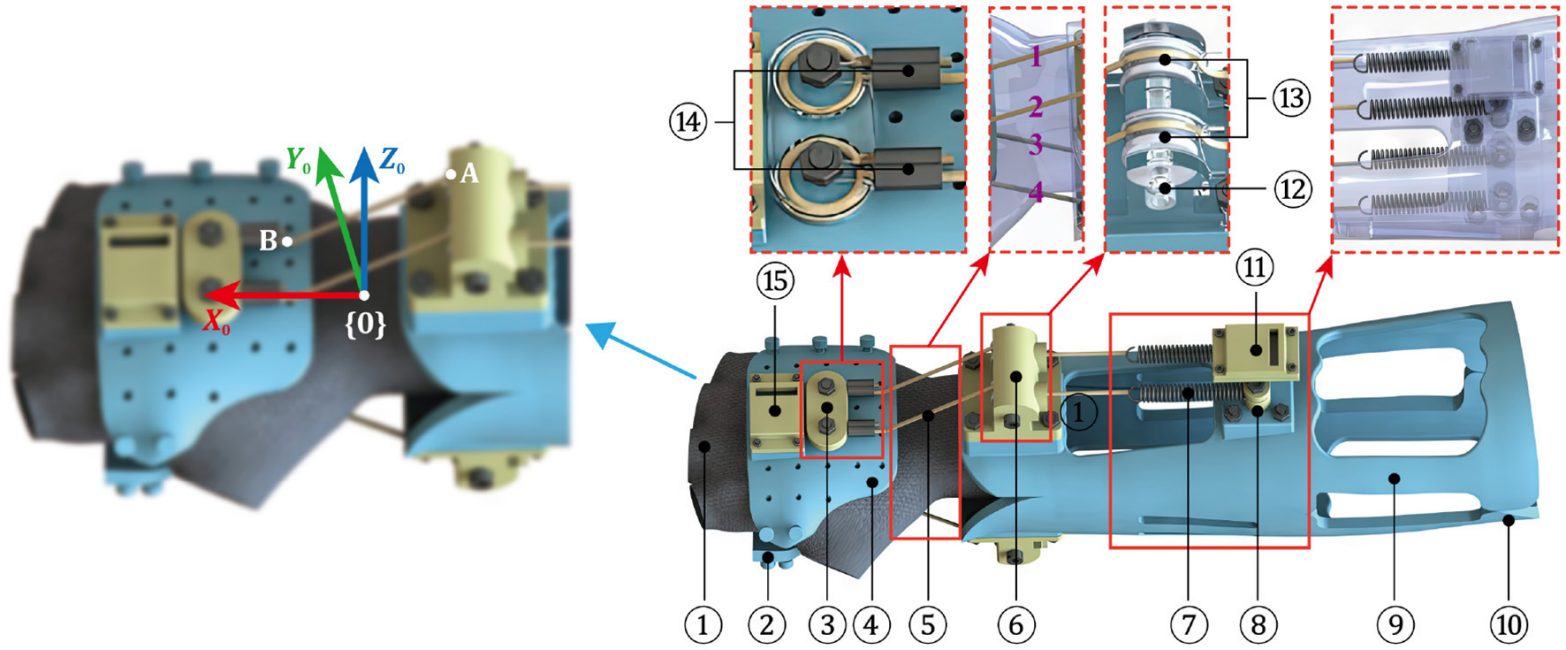
Mechanical design of RTD and the defined D-H coordinate system (1-Flexible half-finger glove; 2-Palamar component of hand module; 3-Winding end; 4-Dorsal component of hand module; 5-Rope-spring kit; 6-Pulley block; 7-Extension spring; 8-Fixed end of spring; 9-Ventral component of the forearm module; 10-Dorsal component of the forearm module; 11-Installation platform of IMU sensor; 12-Pulley bracket; 13-Pulley; 14-Double-hole aluminum sleeve; and 15-Installation platform of IMU sensor).

During the early treatment of rehabilitation, the fractured radius after closed reduction is strictly required to sustain a relatively static condition, thus the significance on fixation performance of the exoskeleton is greatly highlighted. In view of this, the design of AFD primarily adopts rigid structure and parts for ensuring the protection effect of wrist. However, due to the expansion of actual ROM and the impact of resistant force during strengthening exercise, the rigid components might give rise to an unpleasant experience for DRF patient. To solve this problem, a rigid-flexible coupled resistance training device (RTD) was proposed in this work, as shown in Figure 7.

Similar to the design of AFD, the main body of RTD includes a hand module and a forearm module. As to the hand module, notably, a flexible half-finger glove is added and stitched in the internal sides of palmar and dorsal components, which not only provides a rigid connection with the resistance module, but also increases the wearing comfortableness during exercise. More importantly, the previously displayed wrist module in AFD is canceled, instead a resistance module is attached to connect the two wearable modules. The resistance module is composed of four independent rope-spring kits, two of which are installed in the dorsal side of arm, while the others are distributed in the opposite side. One end of the rope-spring kit is fixed in the forearm module, and the other end is connected with the hand module, the direction of it is altered by the pulley mechanism, which also contributes to reduce the friction of rope during expansion and contraction.

Theoretically, which group of rope-spring kits is in application depends on the motion pattern of wrist. For example, the kit 1 and 2 receive pulling force when the DRF patient executes a flexion movement, while the kit 1 and 3 are tensioned if the radial deviation is performed. The resistant force is supposed to be increased along with the expanded motion degree of wrist, meanwhile the rope-spring kits distributed in the contrary side of wrist movement are bent and have no influence on the action. Therefore, by replacing the spring with different stiffness, the desired resistant force of RTD can be obtained.

As discussed before, the improvement of muscle strength can be realized by overcoming an additional torque provoked by the rope-spring kits. The adopted ropes are not easily deformed, hence the resistance force is mainly originated from the springs. As exhibited in Figure 7, the outlet position of rope in pulley mechanism is denoted as point A, and the fixed end of rope on the hand module is point B. In theory, the elongation of spring can be represented by the variation of distance between point A and point B. In this work, the workspace of hand module is analyzed using the modified Denavit-Hartenberg (D-H) method. The base frame {0} is attached in the forearm module, in which the *Z*_0_ axis is coincide with the rotation axis of F/E movement. Here, the motion angles of wrist F/E and R/U are represented by θ_1_ and θ_2_, respectively. The associated D-H parameters for RTD are listed in Table 1.

**Table 1:** Parameters of the modified D-H method for RTD.

***i***	***a*_*i*−1_(mm)**	**α_*i*−1_(rad)**	***d*_*i*_(mm)**	**θ_*i*_(rad)**
1	0	0	0	θ_1_
2	0	−π/2	0	θ_2_−π/2

In general, the homogeneous transformation matrix between two adjacent frames, namely {*i*−1} and {*i*}, is conventionally described as:


(24) $$\begin{array}{c} i-1\\ i \end{array}T=\left[\begin{array}{cccc} C\theta_{i} & -S\theta_{i} & 0 & a_{i-1}\\ S\theta_{i}C\alpha_{i-1} & C\theta_{i}C\alpha_{i-1} & -S\alpha_{i-1} & -S\alpha_{i-1}d_{i}\\ S\theta_{i}S\alpha_{i-1} & C\theta_{i}S\alpha_{i-1} & C\alpha_{i-1} & C\alpha_{i-1}d_{i}\\ 0 & 0 & 0 & 1 \end{array}\right]$$

where *Sθ*_*i*_, *Cθ*_*i*_, *Sα*_*i*_, and *Cα*_*i*_ represent sinθ_*i*_, cosθ_*i*_, sinα_*i*_, and cosα_*i*_, respectively. Therefore, by substituting the D-H parameters of RTD into Equation (24), the transformation matrix $\begin{array}{c} 0\\ 1 \end{array}T$ and $\begin{array}{c} 1\\ 2 \end{array}T$ can be obtained, then the transformation matrix between frame {0} and frame {2} is calculated by:


(25) $$\begin{array}{c} 0\\ 2 \end{array}T=\begin{array}{c} 0\\ 1 \end{array}T_{2}^{1}T=\left[\begin{array}{cccc} C\theta_{1}S\theta_{2} & C\theta_{1}C\theta_{2} & -S\theta_{1} & 0\\ S\theta_{1}S\theta_{2} & S\theta_{1}C\theta_{2} & C\theta_{1} & 0\\ C\theta_{2} & -S\theta_{2} & 0 & 0\\ 0 & 0 & 0 & 1 \end{array}\right]$$

According to the discussion above, the point B of hand module in frame {0} is calculated by:


(26) 0BP=[0BPx0BPy0BPz1]=02TB2P =[Cθ1Sθ2Cθ1Cθ2−Sθ10Sθ1Sθ2Sθ1Cθ2Cθ10Cθ2−Sθ2000001][2BPxPyBPzB1]

In terms of the obtained coordinate value of point B, the distance between A and B after a certain wrist movement is:


(27) $$d_{AB}=\sqrt{{\left(\begin{array}{c} 0\\ B \end{array}P_{x}-\begin{array}{c} 0\\ A \end{array}P_{x0}\right)}^{2}+{\left(\begin{array}{c} 0\\ B \end{array}P_{y}-\begin{array}{c} 0\\ A \end{array}P_{y0}\right)}^{2}+{\left(\begin{array}{c} 0\\ B \end{array}P_{z}-\begin{array}{c} 0\\ A \end{array}P_{z0}\right)}^{2}}$$

while the initial distance between A and B is:


(28) $$d_{0}=\sqrt{{\left(\begin{array}{c} 0\\ B \end{array}P_{x0}-\begin{array}{c} 0\\ A \end{array}P_{x0}\right)}^{2}+{\left(\begin{array}{c} 0\\ B \end{array}P_{y0}-\begin{array}{c} 0\\ A \end{array}P_{y0}\right)}^{2}+{\left(\begin{array}{c} 0\\ B \end{array}P_{z0}-\begin{array}{c} 0\\ A \end{array}P_{z0}\right)}^{2}}$$

then the actual extension value of spring can be expressed as follows:


(29) $$\begin{array}{l} d=d_{AB}-d_{0} \end{array}$$

For RTD, the resistance module includes four rope-spring kits, whose initial endpoint positions of A and B in base frame {0} are given by:


(30) $$\begin{array}{l} A=\left[\begin{array}{c} A_{1}\\ A_{2}\\ A_{3}\\ A_{4} \end{array}\right]=\left[\begin{array}{ccc} -12 & 40 & 12\\ -12 & 40 & -12\\ -12 & -40 & 12\\ -12 & -40 & -12 \end{array}\right] \end{array}$$

(31) $$\begin{array}{l} B=\left[\begin{array}{c} B_{1}\\ B_{2}\\ B_{3}\\ B_{4} \end{array}\right]=\left[\begin{array}{ccc} 42 & 12.5 & 12\\ 42 & 12.5 & -12\\ 42 & -12.5 & 12\\ 42 & -12.5 & -12 \end{array}\right] \end{array}$$

**Figure 8:**
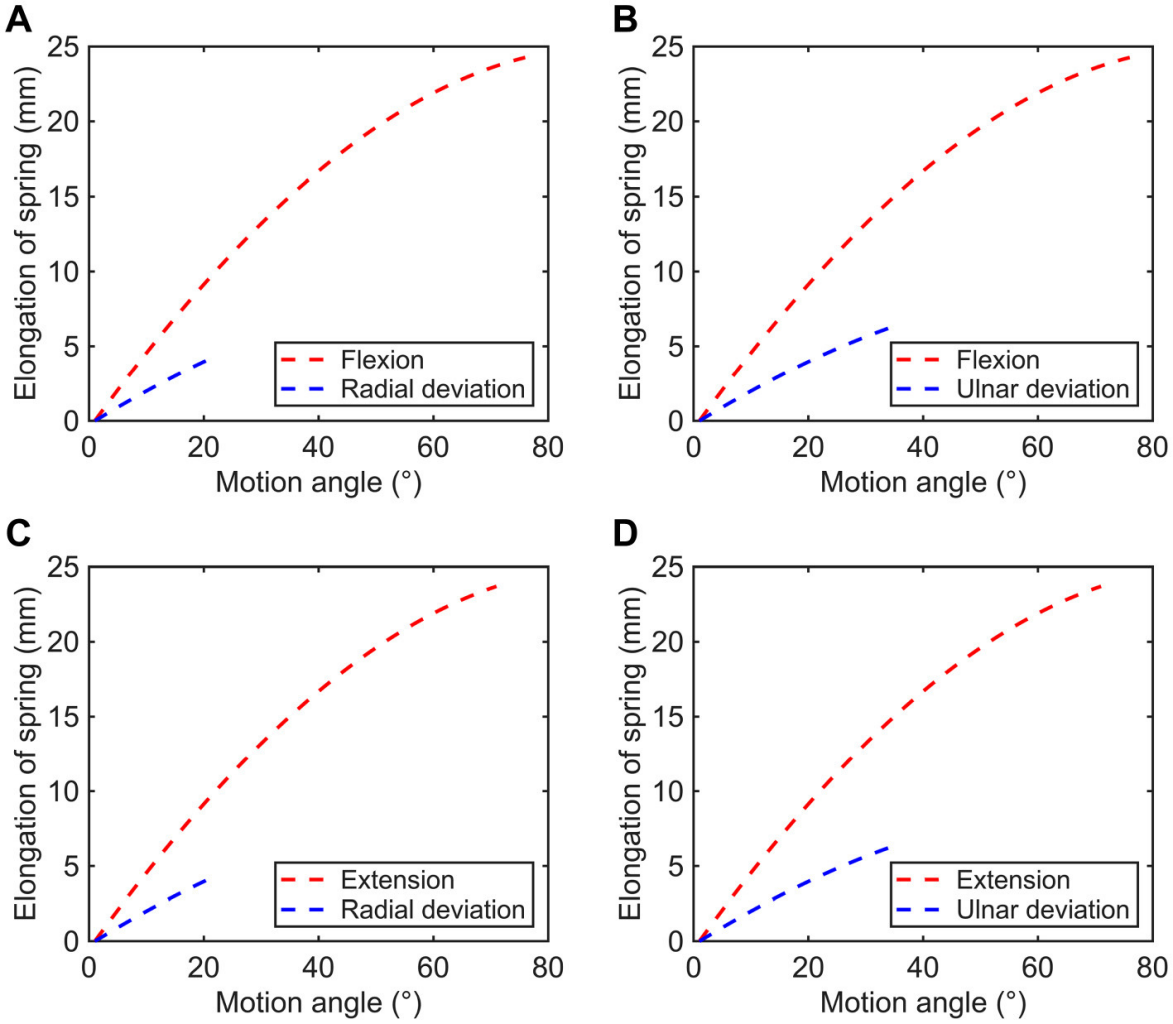
Variations in spring elongation of the rope-spring **(A)** kit 1, **(B)** kit 2, **(C)** kit 3, and **(D)** kit 4.

As mentioned before, the available range of F/E movement $\theta_{1}\in \left[-75^{◦},70^{◦}\right]$, and the range for R/U motion $\theta_{2}\in \left[-20^{◦},35^{◦}\right]$. Based on previous analysis, the elastic deformations of four springs under different motion patterns can be calculated, the relationships between spring elongations and motion angles are exhibited in Figure 8.

In addition, the applied force of each group of two rope-spring kits can be calculated by:


(32) $$\begin{array}{l} F_{\text{Group}}=2kx \end{array}$$

where *x* is the spring elongation, *k* is the spring constant given by:


(33) $$\begin{array}{l} k=\frac{Gd^{4}}{8nD^{3}} \end{array}$$

To investigate the influence of resistance levels on human muscle activity, two spring modules with different stiffnesses were employed in this work. According to Equations (32, 33) and the physical parameters of adopted extension springs listed in Table 2, the available resistant force of RTD for each motion patterns can be obtained. As presented in Table 3, the types of low and high resistance denote the conditions using springs with wire diameters of 0.8 and 1.0 mm, respectively.

**Table 2:** Physical parameters of the adopted extension springs.

**Parameter**	**Value**
Shear modulus, *G*(GPa)	200
Mean diameter, *D*(mm)	10
Wire diameter, *d*(mm)	0.8 and 1.0
Number of the active coils, *n*	25

**Table 3:** Available resistant force for each motion pattern of RTD.

**Type**	**F (N)**	**E (N)**	**R (N)**	**U (N)**
Low resistance	24.26	23.70	4.15	6.49
High resistance	48.52	47.40	8.30	12.97

### 3.3 Data acquisition system

**Figure 9:**
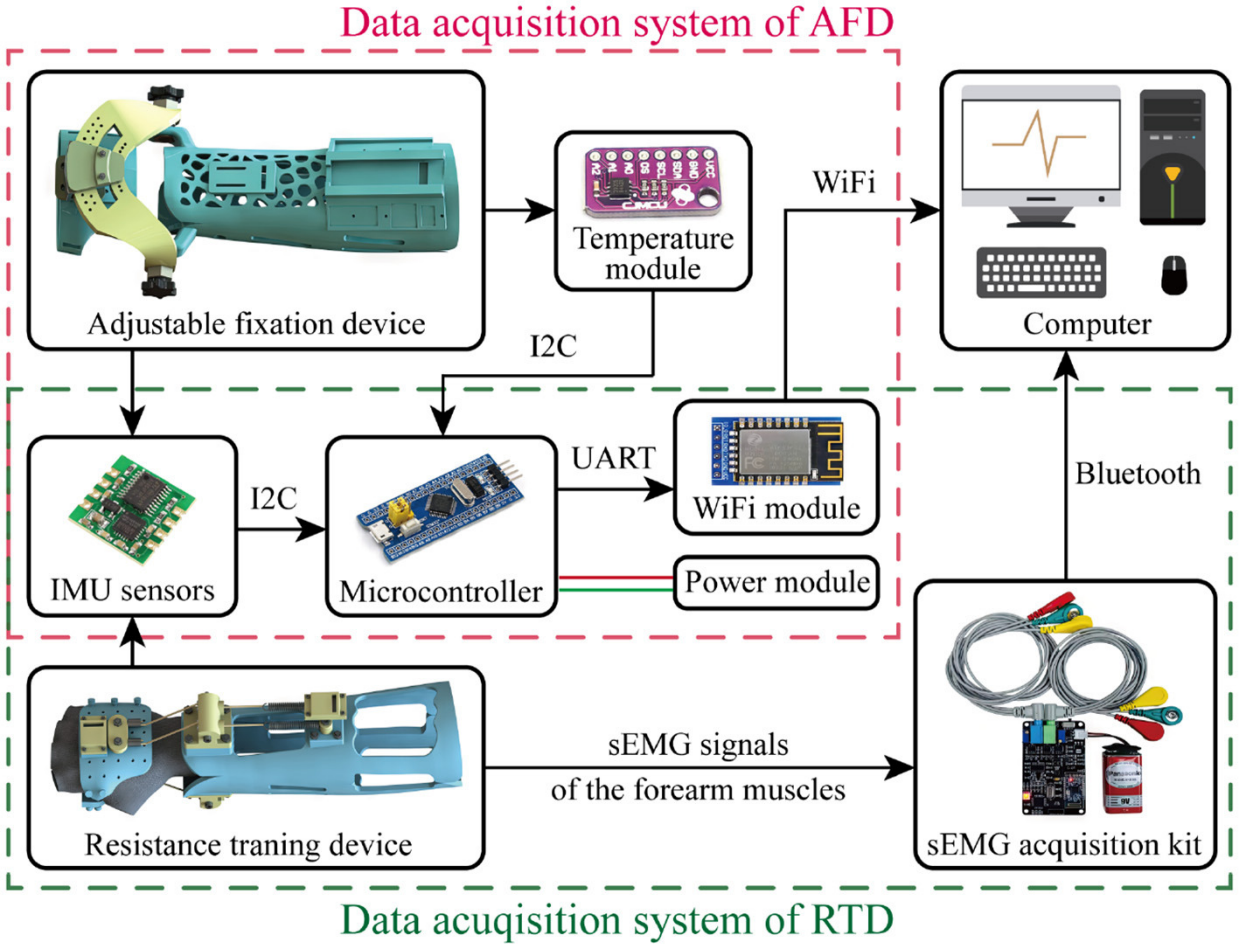
Hardware architectures of the data acquisition systems for AFD and RTD.

As presented in Figure 9, the two exoskeleton devices are combined with respective data acquisition systems to evaluate their practical function. Notably, AFD and RTD are both equipped with two posture sensors, respectively distributing in the aforementioned installation platforms of hand and forearm modules. The required fixation for DRF could last a long time, thus a temperature module is attached in the forearm module of AFD, for monitoring the temperature variation of patient wearing the exoskeleton. The corresponding joint angle and temperature data are transmitted into a STM32 microcontroller, which is an information processing and exchanging center that communicated with computer based on a WiFi module. As to the RTD, the resistant effect is examined by an additional sEMG acquisition device, in which the electrodes (i.e., the red, green, and yellow ones) should be placed in the outer skin of forearm for bioelectric signal detection. The computer serves for data collection and visualization, the relevant joint angle and the cumulative exercise times will be calculated and displayed in real time, resulting to more intuitive observation for the patient and more quantitative supervision for the attending doctor.

## 4 Experiments and functional evaluation

Based on the design schemes presented above, the prototypes of AFD and RTD were established for function evaluation. The main bodies of these two exoskeleton devices, including the hand, wrist and forearm modules, were fabricated by 3D printing with the material of UV curable resin. As suggested in previous researches (Li et al., 2023), the joint motion and muscle strength are two indispensable indexes to track human states. To investigate the application performance of exoskeleton device on human movement, physical experiments were carried out on three healthy subjects (Subject 1: female, 45 kg, 1.62 m, 24 years old; Subject 2: male, 65 kg, 1.72 m, 30 years old; Subject 3: female, 48 kg, 1.67 m, 29 years old). The experimental protocol has been approved by the Ethics Committee of Zhongda Hospital Southeast University, and the subjects have given informed consent to participate in the experiments by signing a written agreement.

**Figure 10:**
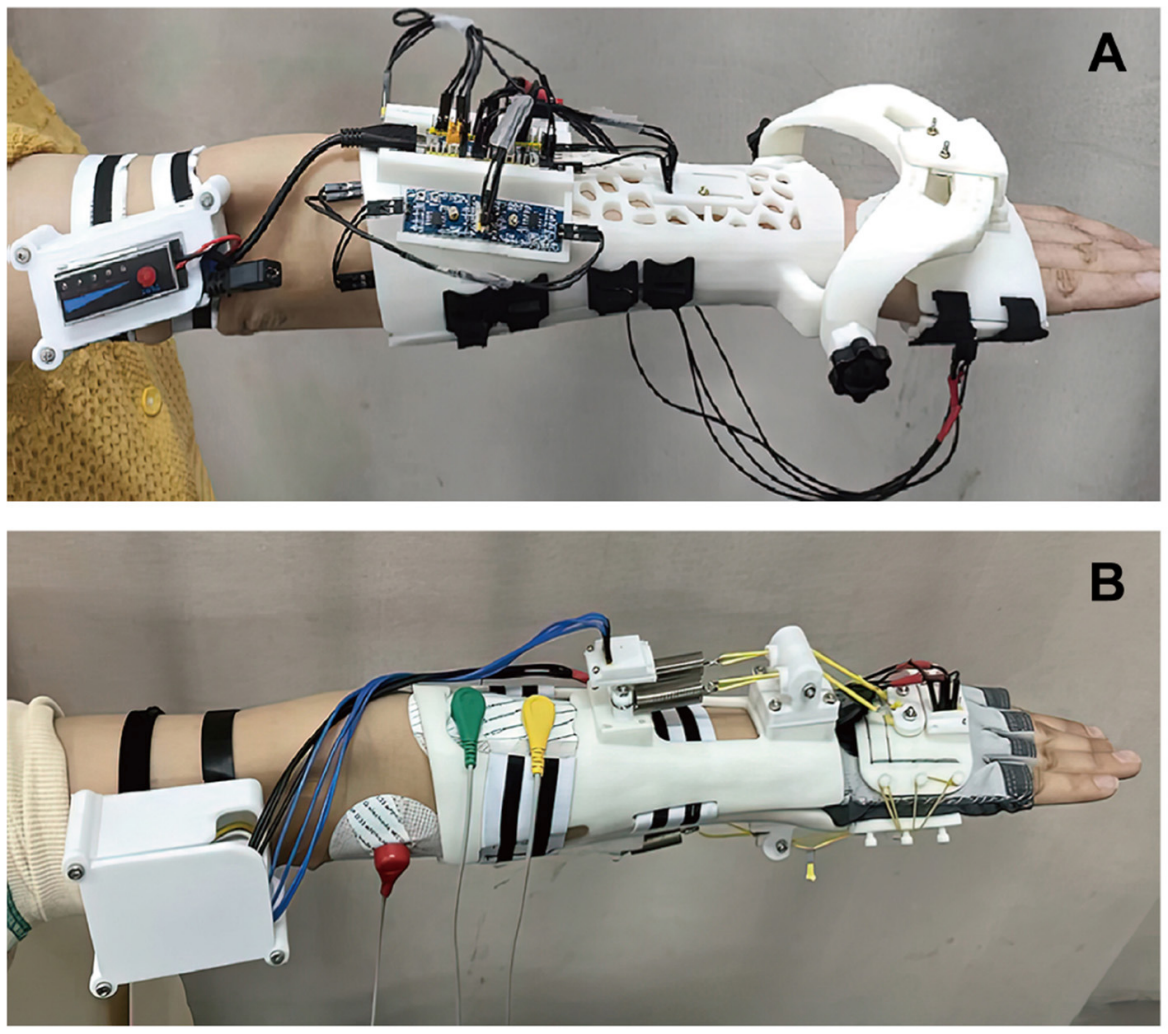
Experimental conditions and wearing appearances of **(A)** AFD and **(B)** RTD.

As presented in Figure 10A, the hand and forearm modules are worn by Subject 1 and the paired components are tied with Velcro types. Most electronic elements of the required data acquisition system for AFD are deployed to attach in the installation platforms, while some of them are carried by arm, including the portable battery module. To validate the feasibility in detecting and recording the motion ranges of different postures, several experiments under wrist, elbow and shoulder movements were systematically implemented. Notably, the key of wrist experiment focuses more on the reachable range of AFD, while the experiments of elbow and shoulder joint are mainly concentrated in the counting precision of exercise times.

The experimental conditions of RTD and the attachment of surface electrodes are exhibited in Figure 10B, where the red electrode serves as a signal reference, while the green and the yellow ones are applied for the actual bioelectric signal detection and placed on the corresponding skin region of muscle. To study the actual impact of RTD on muscle activities in different individuals, all three subjects participated in the resistance training experiments were equipped with RTDs customized to their forearm contours.

### 4.1 Attitude monitoring

**Figure 11:**
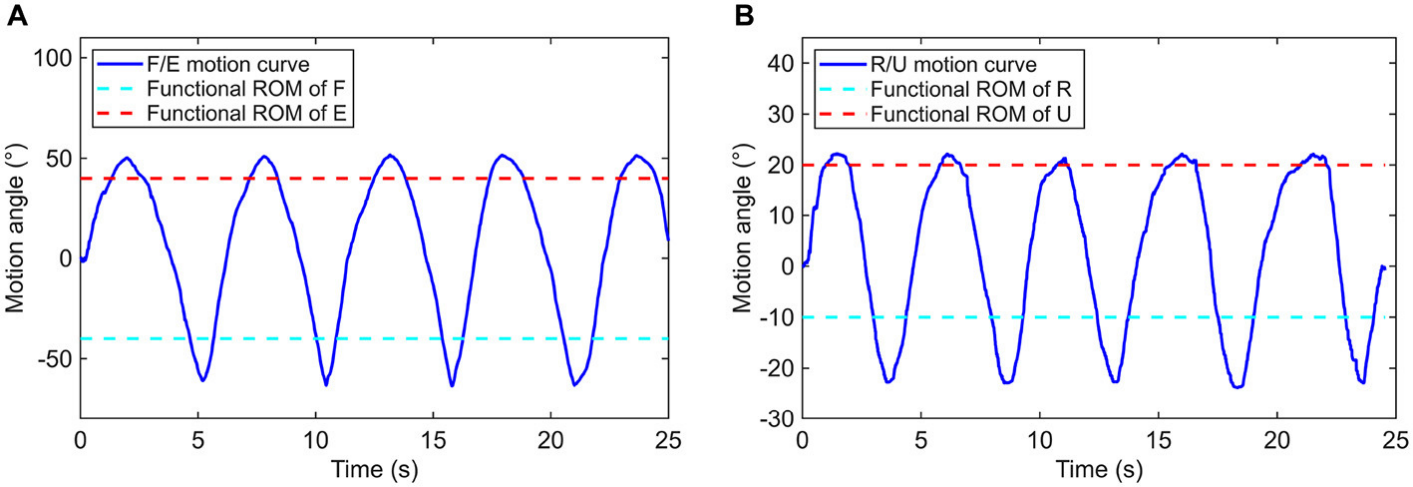
Motion curves based on **(A)** flexion/extension and **(B)** radial/ulnar deviation of the wrist joint.

In this work, the two groups of wrist movement were respectively performed for five consecutive times by experimenter, the posture data is recorded and the actual range of each action was calculated. As illustrated in Figure 11, the motion angle equals to zero when the wrist is at its neutral position, extension and ulnar deviation are defined as the positive direction, as opposite to the flexion and radial deviation. According to motion curves, it is apparent that the accessible ranges of AFD for flexion/extension and radial/ulnar deviation are all capable of covering the functional ROMs of wrist joint.

**Figure 12:**
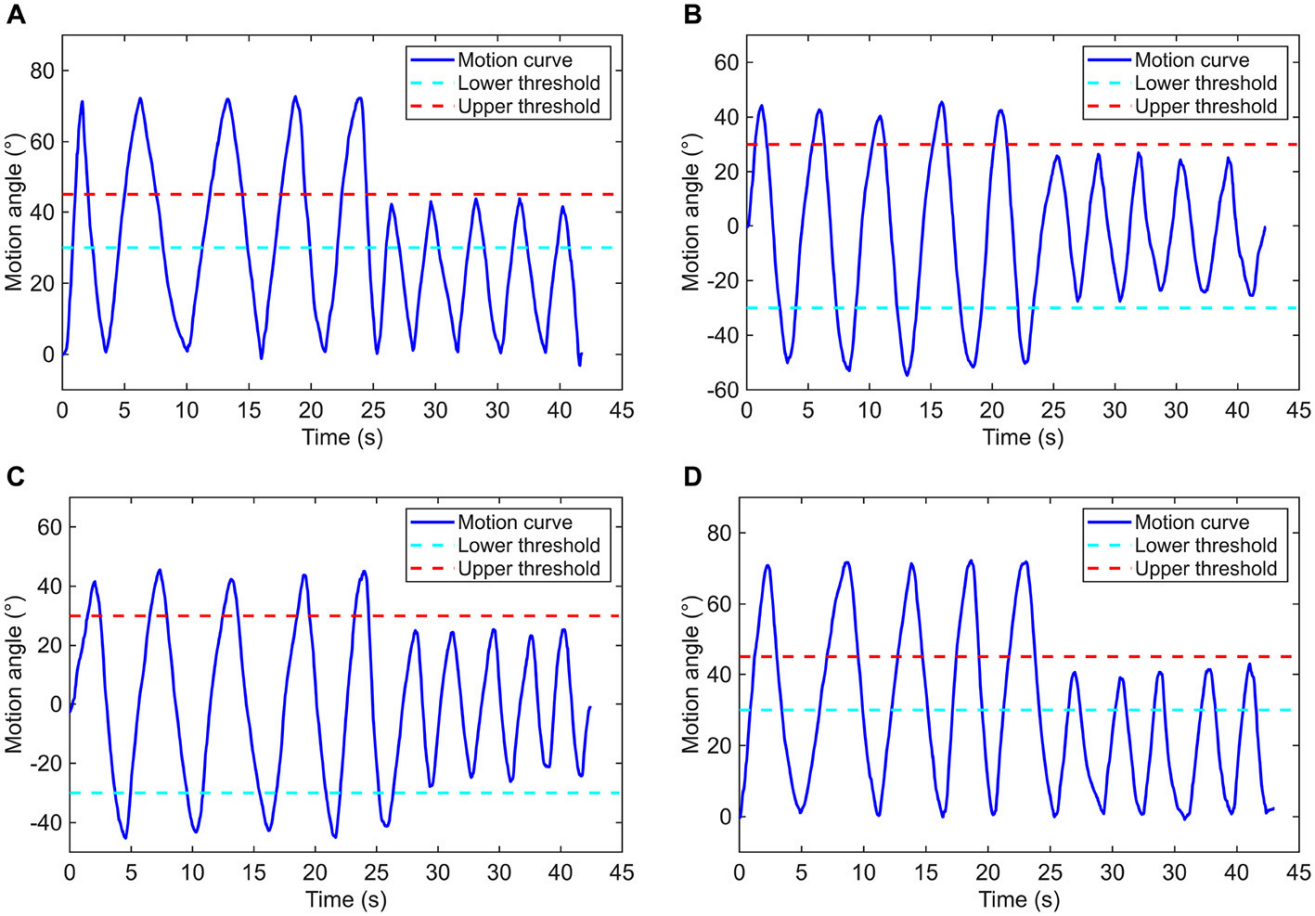
Motion curves based on **(A)** flexion/extension of the elbow joint, **(B)** abduction/adduction, **(C)** flexion/extension, and **(D)** internal/external rotation of the shoulder joint.

As for the elbow and shoulder joints, different thresholds were defined for the judgment on whether the basic range requirement of a motion behavior is satisfied or not. The employed thresholds for each motion patterns of elbow and shoulder are listed in Table 4. In this work, a continuous movement that successively exceeds the upper threshold and the lower threshold is regarded as one time of complete training action. To estimate the counting precision and sensitivity on training times, the experiments were deliberately configured by establishing conditions in which half of the total repetitive motions were correctly counted, while the other half were not. As illustrated in Figure 12, 10 times of reciprocating motion for each pattern have been conducted. According to the experimental results of each motion pattern, only the first five times of action can be effectively counted, while the last five are failed. The results are as expected, hereby the monitoring function of elbow and shoulder postures AFD is demonstrated to be reliable.

**Table 4:** Range thresholds for each motion pattern of elbow and shoulder joint.

**Joint name**	**Motion pattern**	**Upper threshold (°)**	**Lower threshold (°)**
Elbow	Flexion/extension	45	30
	Abduction/adduction	30	–30
Shoulder	Flexion/extension	30	–30
	Internal/external rotation	45	30

In contrast to previous studies that mainly focused on immobilizing fractured wrists using plaster splint or other fixation devices, the AFD introduces a novel, adjustable wrist exoskeleton mechanism. It provides stable protection for DRF while allowing limited joint movements to reduce muscle stiffness. Meanwhile, the AFD also offers effective motion data for rehabilitation exercises, contributing to better quantification of the active rehabilitation process in DRF patients.

### 4.2 Resistant influence

The neuromuscular system of a human body includes plenty of muscles and nerves, every movement of the body is realized based on the communication from the brain to muscles. The muscular system is comprised of several specialized cells named muscle fibers, and the predominant function of them is for contractility. In general, the activities of muscle fiber can be detected and digitalized by the electromyography (EMG). As one of the EMG technology, surface electromyography (sEMG) has been extensively applied in wearable technologies (Xiao et al., 2024) for clinical diagnosis and functional supervision of muscle rehabilitation.

Here, the contraction activities of extensor carpi ulnaris (ECU) and flexor carpi ulnaris (FCU) during F/E movement, flexor carpi radialis (FCR) and flexor carpi ulnaris (FCU) during R/U movement, were systematically investigated. ECU is a forearm muscle extending from the lateral epicondyle of the humerus to the posterior border of the ulna, it plays a crucial role in wrist extension. FCU is a flexor muscle that originates from the medial epicondyle of the humerus to the posterior border of the ulna, it is mainly associated with wrist flexion and ulnar deviation. FCR is emanated from the medial epicondyle of the humerus and extends distally to the base of the second metacarpal bone, it contributes to radial deviation of the wrist, facilitating movement toward the thumb side. The selected three muscles are distributed in a superficial layer of forearm, and the bioelectric signals of this superficial muscle are more effectively and evidently to be detected on the skin. The sEMG signals of muscles under different experimental conditions were monitored in real time based on a sEMG acquisition kit presented in Figure 9.

**Figure 13:**
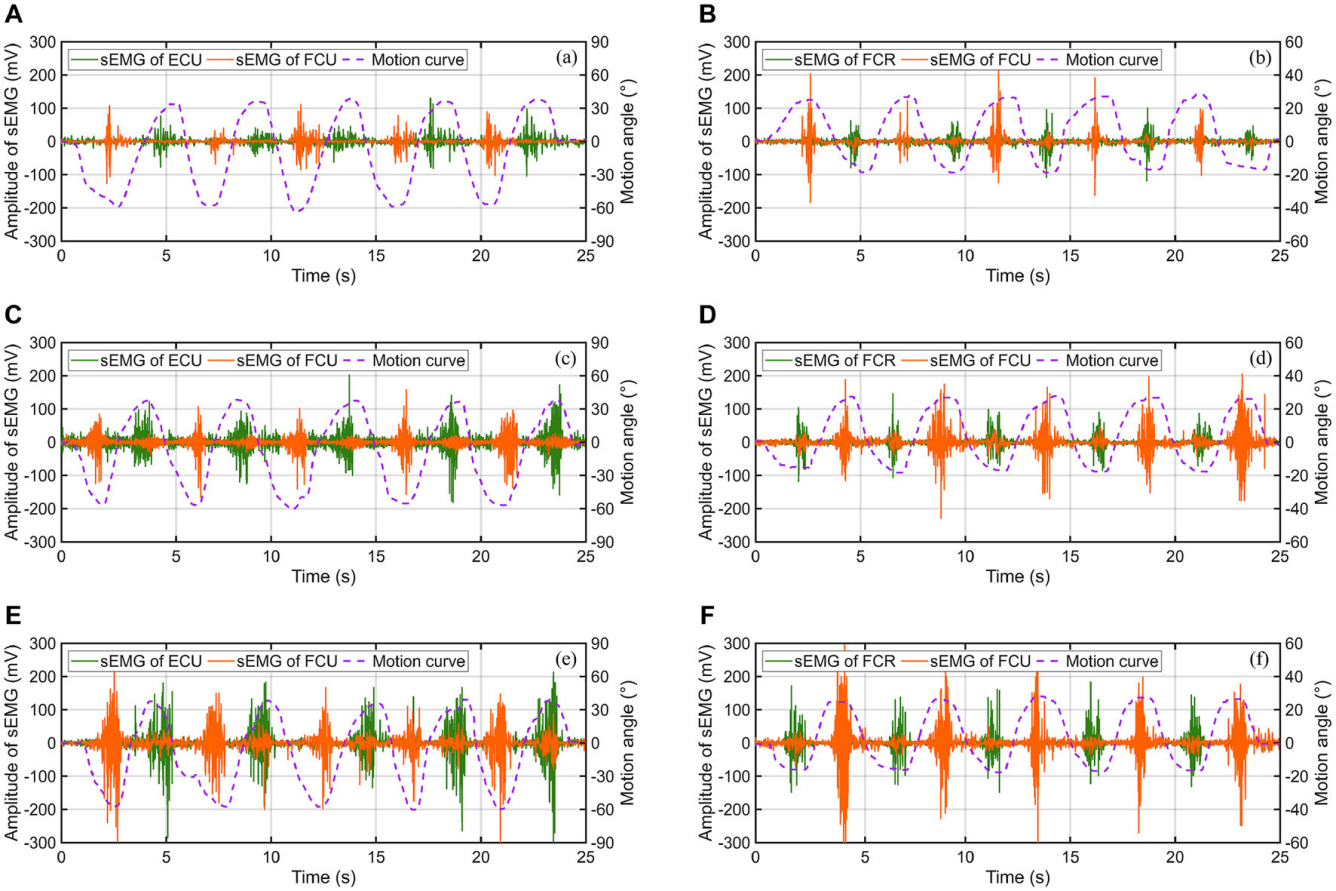
The sEMG signals of muscles under different motion patterns and experimental conditions: **(A)** F/E movement without resistance, **(B)** R/U movement without resistance, **(C)** F/E movement with low resistance, **(D)** R/U movement with low resistance, **(E)** F/E movement with high resistance, and **(F)** R/U movement with high resistance.

For each experimental subject, the F/E and R/U movement of wrist under the condition without any resistance, the condition with low resistance and the condition with high resistance were required to be independently performed for five consecutive times. Compared with the early stage, the ROM for resistance training is limited to 55° for wrist flexion and 35° for wrist flexion, while radial deviation is limited to 15° and ulnar deviation to 25°. However, the directly detected bioelectric data may contain a significant amounts of noise and artifacts, which can negatively affect signal interpretation (Yin et al., 2024). Algorithmic intervention served as a popular strategy to reduce such distortions and enhance signal clarity. Here, the sEMG signals were further filtered by the wavelet transform algorithm, with the basis function of Daubechies (db4), the decomposition level of 2, and a sampling rate of 1,000 Hz. Wavelet transform offers an effective denoising method for electromyographic signals due to its time-frequency localization and multi-scale resolution, it removes noise while preserving signal details, enhancing signal-to-noise ratio and facilitating accurate analysis of EMG activity patterns. The eventually acquired sEMG signal data of one of the subjects are presented in Figure 13.

The integrated EMG (iEMG), a frequently-used feature extraction methods of sEMG signals, was employed as an evaluation index of the muscular contraction level. As described in Equation (34), iEMG is the mathematical integration of rectified EMG signals over a specified period of time, where *T* denotes the length of integration time window, and *Data*[*t*] is the EMG signal.


(34) $$\begin{array}{l} \text{iEMG}=\int_{0}^{T}|Data\left[t\right]|dt \end{array}$$

**Figure 14:**
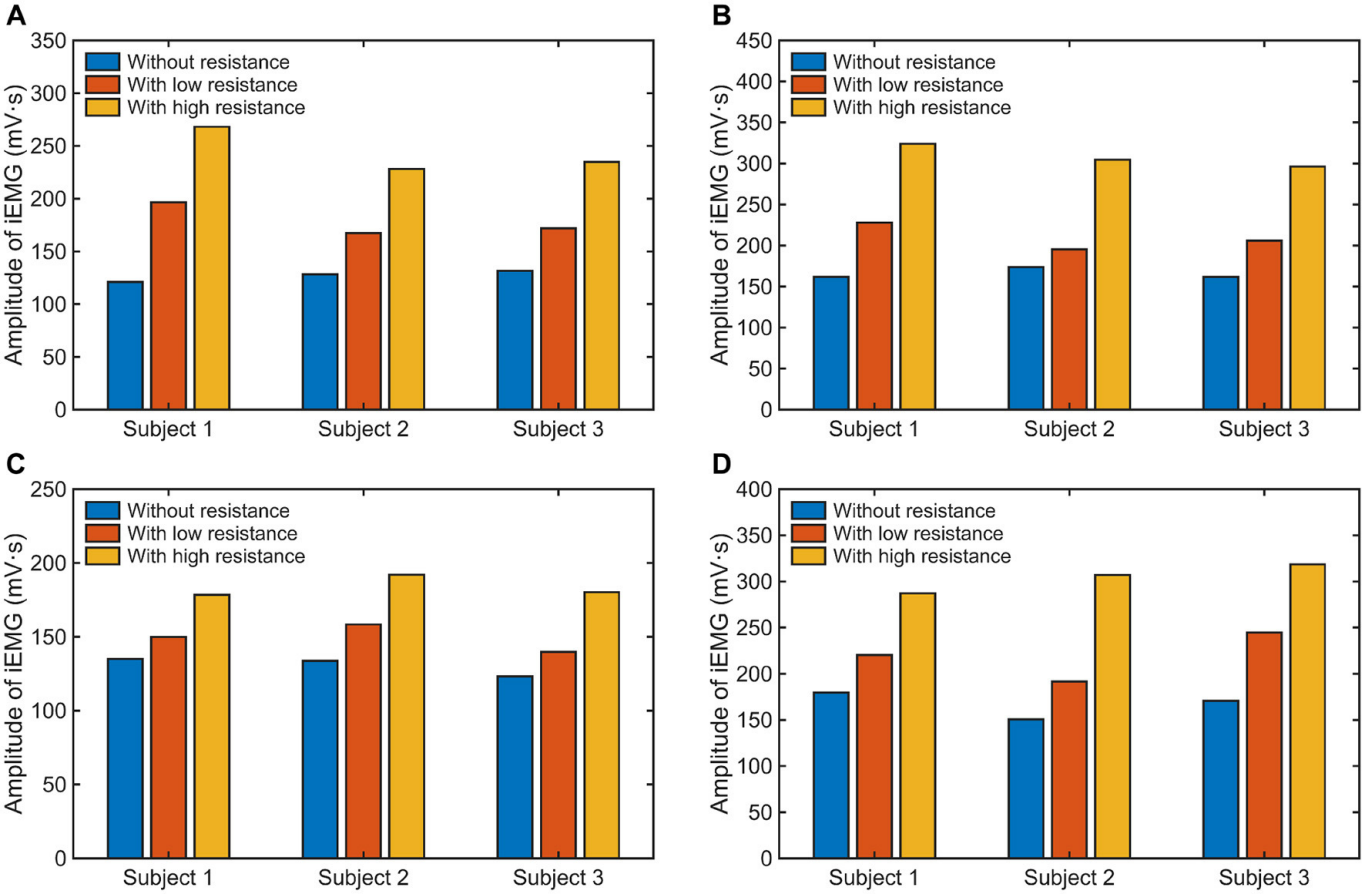
The iEMG values of different muscles: **(A)** FCU under F/E movement, **(B)** ECU under F/E movement, **(C)** FCR under R/U movement, and **(D)** FCU under R/U movement.

**Figure 15:**
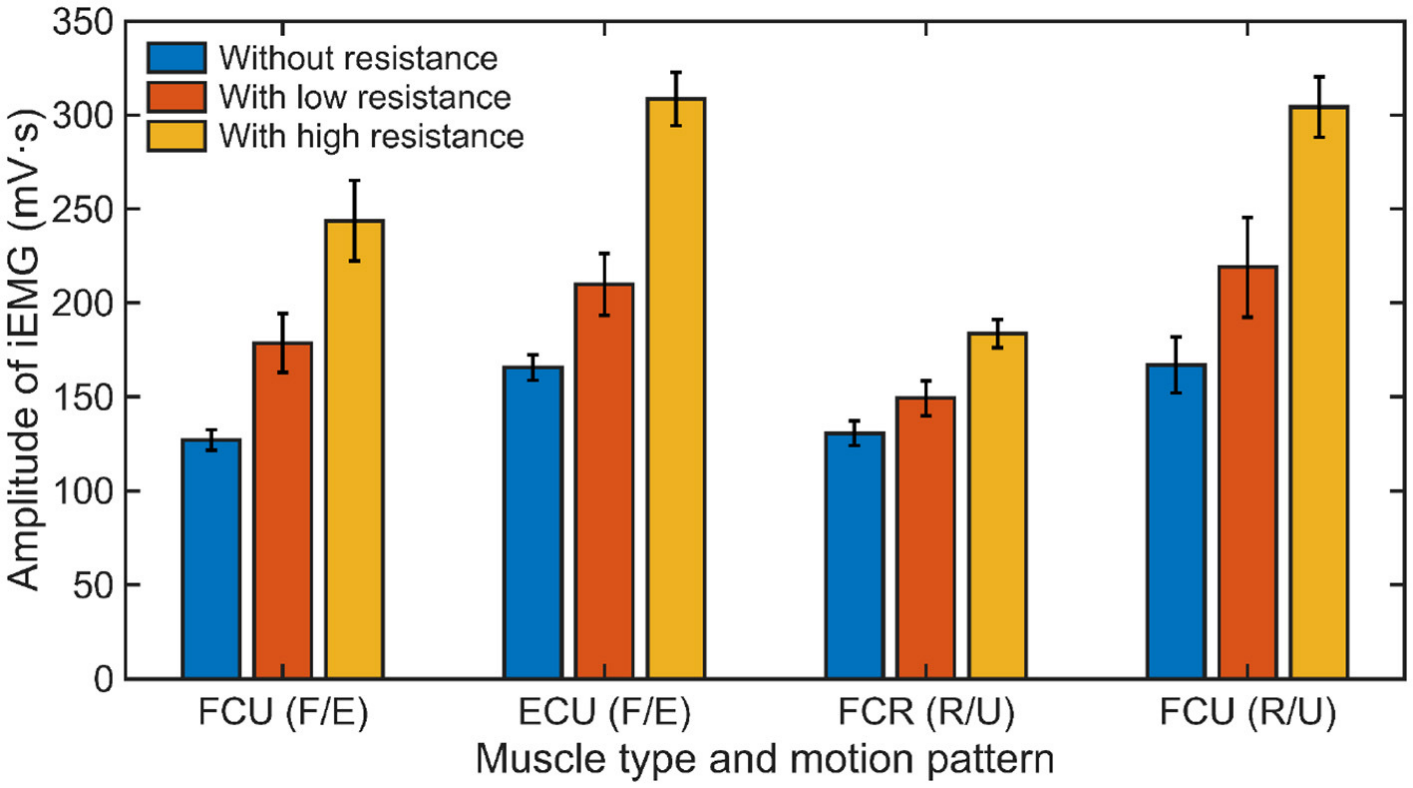
The average iEMG values of all subjects (Mean ± 1SD, *N* = 3) under different experimental conditions.

The calculated iEMG values of different muscles under three experimental conditions are shown in Figure 14. The results demonstrate an apparent growth of muscle activation with the increase of external resistance applied in each motion pattern for all subjects. As depicted in Figure 15, compared to the experimental condition without any constraint, the iEMG values exhibit distinct patterns under varying resistance levels. Specifically, during the F/E movement, the iEMG values increase by approximately 35% and 22% for FCU and ECU under low resistance, respectively, and by approximately 91% and 86% under high resistance, respectively. Similarly, during the R/U movement, the iEMG values increase by approximately 12% and 31% for FCR and FCU under low resistance, respectively, and by approximately 37% and 82% under high resistance, respectively.

According to the experimental results of RTD, the application of an resistant force is essential for the patients with diverse muscular strength. Compared to previous studies that mainly focused on the passive motion of wrist joint under the assistance of exoskeleton robots, the RTD proposed in this study aims to enhance the active rehabilitation ability of DRF patients, which is of great significance for helping them regain normal function of wrist movement. Notably, the springs employed on the RTD can be freely replaced with ones of different stiffnesses according to the baseline muscle strength of patients. For patients with weak muscle strength (e.g., old patients or the patients in the early stage of rehabilitation), it is recommend that the RTD be equipped with low resistance. To continue gaining benefits from strength enhancing activities, the resistance force is required to be progressively increased.

In this section, the application performance of AFD and RTD for wrist rehabilitation were experimentally evaluated. The research focus lies in validating the joint monitoring function and exploring the resistance training influence of exoskeleton devices. Therefore, although the data were obtained from healthy human subjects, the summarized principles related to joint motion and resistance training can be generalized to patients suffering from DRFs with varying characteristics. However, despite the validated advantages, there are still some limitations to the current exoskeleton devices. Due to the physiological differences between healthy individuals and fracture patients, further integration of the exoskeleton devices with force/torque sensors is conducive to gain a deeper understanding of the wrist dynamics in DRF patients. Moreover, the exoskeleton device can be further integrated with additional mechanisms to automatically impose physical intervention that limit joint movement when the monitored force/torque data reach safety thresholds defined by doctors based on clinical experience during joint motion. Meanwhile, there are also some potential issues that could affect the application of exoskeleton devices. In clinical practice, patients may experience issues such as poor comfort and compliance when wearing exoskeleton devices for extended periods. During the long-term application of the exoskeleton devices, it is necessary to develop more systematic safety protocol and monitoring plan in conjunction with the diagnoses of doctor.

## 5 Conclusion

In this work, biomechanical models of the human wrist affected by DRF were established firstly, then the auxiliary effect of external fixation on the fractured bones was demonstrated, and the mechanism of external resistance on the wrist movement was revealed. According to the biomechanical analysis, two portable and wearable exoskeleton devices were proposed to facilitate the long-term rehabilitation of DRF. The adjustable fixation device (AFD) provides the external protection and range-limited mobilization of wrist in the early stage, while the functional recovery of relevant muscles is achieved by the resistance training device (RTD) in the later stage. Based on physical experiments, the actual motion ranges of AFD were investigated, and the feasibility in monitoring the motion angles of different joints including the wrist, elbow and shoulder were validated. Under free conditions and two levels of resistance, the activities of wrist-related muscles were evaluated using the collected sEMG signals. The calculated iEMG results indicated that the training-induced muscle activation generally increases with the increment in resistance force, which contributes to the enhancement of muscle strength.

To improve the wearing appearance and comfortableness of the proposed exoskeleton devices, our future work will concentrate in upgrading the integration of mechatronic system. Additionally, we will further combine force/torque sensors to monitor the dynamic conditions of fractured wrist for interaction safety during joint movement, and use machine learning methods to evaluate the effectiveness of exoskeleton devices for long-term DRF rehabilitation in a wider range of patients.

## Data Availability

The original contributions presented in the study are included in the article/supplementary material, further inquiries can be directed to the corresponding author.
